# Co-trimoxazole induced hyperkalemia and potassium monitoring in hospitalized patients

**DOI:** 10.1007/s11096-020-01052-x

**Published:** 2020-05-11

**Authors:** Milan M. E. A. Plantaz, Bart A. J. Veldman, Anne C. Esselink, Hanneke W. H. A. Fleuren, Cornelis Kramers

**Affiliations:** 1 Department of Clinical Pharmacy A16, Canisius Wilhelmina Hospital, PO Box 9015, 6532 SZ Nijmegen, The Netherlands; 2 Department of Internal Medicine, Canisius Wilhelmina Hospital, Nijmegen, The Netherlands; 3 Department of Pharmacology-Toxicology, Radboud University Medical Centre, Nijmegen, The Netherlands

**Keywords:** Adverse effect, Co-trimoxazole, Hyperkalemia, Serum potassium, Trimethoprim

## Abstract

*Background* Co-trimoxazole is an antibiotic combination used for the treatment of *Pneumocystis jirovecii* pneumonia, amongst others. Co-trimoxazole is known to increase serum potassium. For this reason, Dutch guidelines advise serum potassium monitoring in high-risk patients. *Objective* This study aimed to determine average serum potassium rise after administration of intravenous co-trimoxazole in hospitalized patients, compared to intravenous ceftriaxone. This study also aimed to determine adherence to Dutch guidelines by measuring the incidence of serum potassium monitoring in these patients. *Setting* Five departments of the Canisius Wilhelmina Hospital, a teaching hospital in Nijmegen, the Netherlands. *Method* Data was collected and compared from patients that received intravenous co-trimoxazole (n = 66) and intravenous ceftriaxone (n = 132) in the period of November 2008–November 2017. For each patient using co-trimoxazole, two patients using ceftriaxone were included in a paired fashion. Baseline and follow-up potassium were collected, if available. Additionally, it was tested if serum potassium was measured around the initiation of antibiotic therapy. *Main outcome measure* Changes in serum potassium where obtainable in 30 patients using cotrimoxazole and 40 patients using ceftriaxone. When compared to ceftriaxone, administration of intravenous co-trimoxazole was associated with a significant mean increase in serum potassium (+ 0.55 mmol/l, 95% CI 0.29–0.80, *p* < 0.001). After correction for confounders (baseline potassium, estimated glomerular filtration rate 30 to < 60, the presence of haematological malignancies and the usage of corticosteroids), this effect shrunk noticeably, but remained significant (+ 0.28 mmol/l, 95% CI 0.03–0.53, *p* = 0.031). *Results* The incidence of hyperkalemia at follow-up was 20% in the cotrimoxazole group, compared to 5% in the ceftriaxone group. Despite this, serum potassium was often not measured in patients using intravenous cotrimoxazole, being 76% at baseline and 55% in the period of 48–120 h after antibiotic therapy initiation, compared to 87% and 34% in the ceftriaxone group respectively. *Conclusion* Adherence to Dutch guidelines was poor as serum potassium monitoring was often not performed. As intravenous co-trimoxazole usage is associated with a significant increase in mean serum potassium, monitoring is strongly recommended.

## Impacts on practice

- Intravenous co-trimoxazole is associated with a significant average increase in serum potassium as compared to intravenous ceftriaxone.
- Serum potassium is frequently not measured before and during intravenous co-trimoxazole therapy, including those with a high risk for hyperkalemia. This study shows the need for potassium measurements during intravenous treatment with co-trimoxazole

## Introduction

Co-trimoxazole (trimethoprim-sulfamethoxazole) is the drug of choice for the treatment of *Pneumocystis jirovecii* pneumonia (PJP), and is also used for certain urinary tract, gastro-intestinal and skin infections [1]. Trimethoprim increases serum potassium concentration by inhibition of the epithelial sodium channel (ENaC) in the distal nephron. This leads to a reduction in sodium reabsorption, and a subsequent decrease in serum potassium excretion [2, 3]. Hyperkalemia may present with symptoms such as muscle pain and nausea. In severe cases, hyperkalemia can result in palpitations, arrhythmias and even death [4]. The first studies describing hyperkalemia in patients receiving co-trimoxazole were published in the 1980s [5]. In a case control study it was found that patients on Renin–Angiotensin-System inhibitors who started co-trimoxazole, had an increased risk of death compared to other antibiotics, probably due to hyperkalemia [6].

Current Dutch guidelines state that serum potassium should be monitored during co-trimoxazole treatment in patients who are at a high risk for hyperkalemia [7, 8]. The Royal Dutch Pharmacists Association guideline states: ‘Serum potassium monitoring is required for patients using co-trimoxazole in combination with potassium sparing diuretics, angiotensin converting enzyme inhibitors (ACEi) or angiotensin receptor blockers (ARBs).’ [7] The Dutch Pharmacotherapeutic Compass mentions: ‘All patients with risk factors for hyperkalemia should be monitored for hyperkalemia, including, amongst others, patients > 70 years, patients with diabetes or heart failure, patients that use other potassium-elevating medication and patients who receive high doses of the drug for the treatment of PJP.’ [8] Even though co-trimoxazole is mostly prescribed orally, it is also prescribed intravenously, mainly for hospitalized patients who suffer from severe PJP and for those who cannot take the drug orally. Generally, patients who receive intravenous co-trimoxazole are sicker and more vulnerable than patients on oral treatment. The serum potassium rising effect of co-trimoxazole is in hospitalized patients that receive the drug intravenously is presently unknown.

## Aim of the study

This study aimed to determine average rise in serum potassium in hospitalized patients receiving intravenous co-trimoxazole, as compared to hospitalized patients receiving intravenous ceftriaxone. It also aims to verify if the current guidelines regarding serum potassium monitoring are being met.

## Ethics approval

The local Canisius Wilhelmina Ziekenhuis (CWZ) ethical committee approved this study under identification number CWZ-064-2019. This study is not subject to the Dutch WMO-law regarding scientific-medical research concerning humans (wet Wetenschappelijk Medisch Onderzoek met mensen). This study has been registered in the Dutch trial database under study identification number NTR7608.

## Methods

**Fig. 1:**
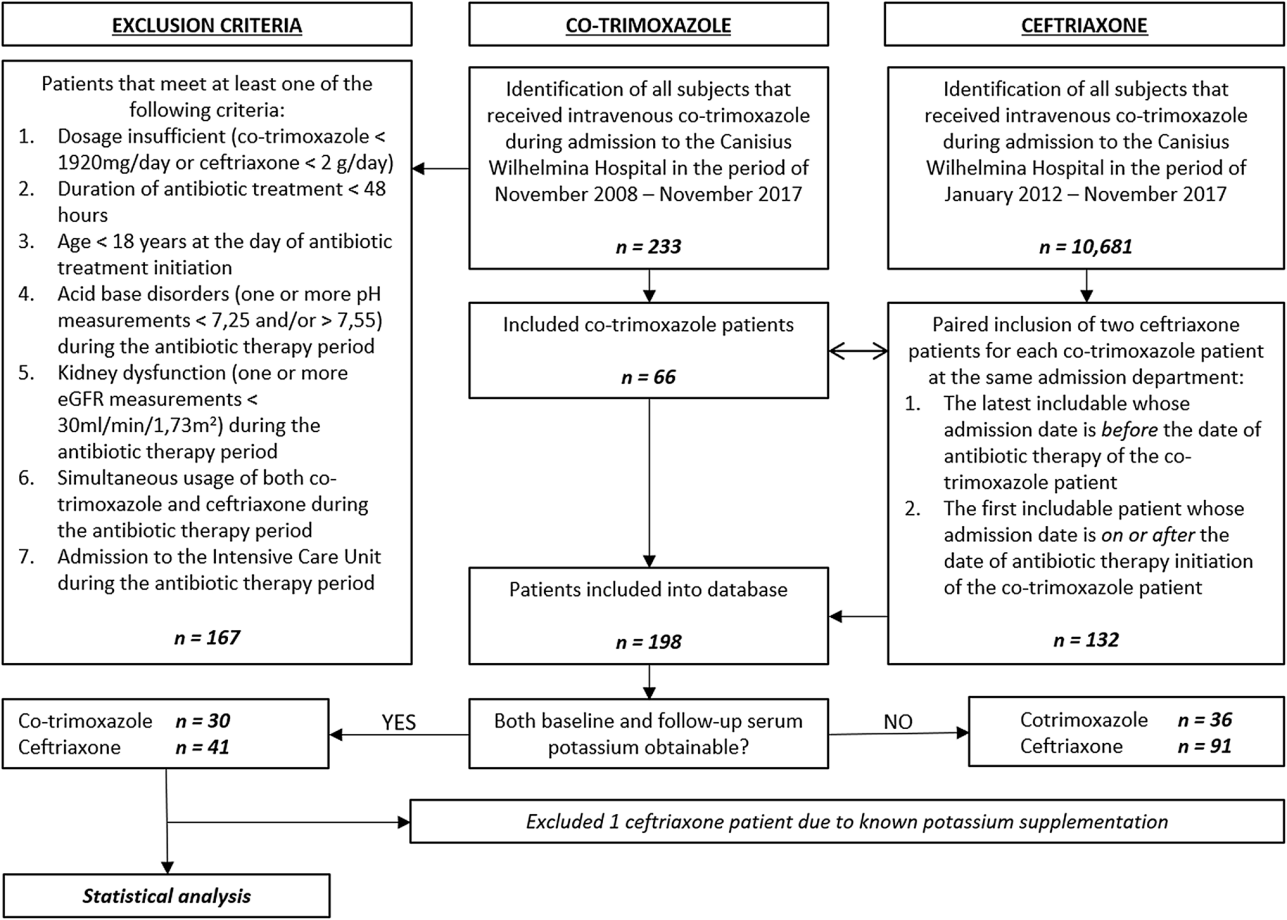
Flow chart of inclusion and exclusion criteria

This study was conducted at the CWZ, Nijmegen, The Netherlands. The CWZ ethical committee approved this study. All patients receiving intravenous co-trimoxazole during admission in the period of November 2008–November 2017 were identified retrospectively. Data were collected using electronic patient files. Patients that met one of the exclusion criteria were excluded: dosage insufficient (< 1920 mg/day), duration of antibiotic treatment < 48 h, age < 18 years, acid–base disorders (pH < 7.25 or > 7.55), kidney dysfunction [defined as estimated glomerular filtration rate (eGFR) < 30 ml/min/1.73 m^2^ and calculated using the Modification of Diet in Renal Disease (MDRD) formula], simultaneous usage of co-trimoxazole with ceftriaxone and admission to the intensive care unit. Patients using ceftriaxone were used as controls, since these are also sick patients needing intravenous antibiotic therapy, and ceftriaxone has no known effect on serum potassium. Paired inclusion of ceftriaxone patients based on admission department and starting date of antibiotic therapy was performed. For each co-trimoxazole patient, two ceftriaxone patients admitted at the same department were included. Both the latest includable patient whose admission date was before the start of antibiotic therapy date of the co-trimoxazole patient, as well as the first includable patient whose admission date was on or after the start of antibiotic therapy date of the co-trimoxazole patient, were included. Exclusion criteria for ceftriaxone patients were the same as mentioned above (insufficient dosage being < 2 g/day for ceftriaxone instead of < 1920 mg/day used for co-trimoxazole). Co-trimoxazole patients from 2009, 2010 and 2011 were coupled to ceftriaxone patients from 2012, 2013 and 2014 respectively due to lack of sufficient ceftriaxone patients from the years 2009–2011. So, for example, a cotrimoxazole patient that started antibiotic treatment on June 11th, 2010, was coupled as if it started antibiotic treatment on June 11th, 2013. In Fig. 1, this process is visualized.

The primary endpoint of this study was change in serum potassium (ΔK: follow-up minus baseline serum potassium. Baseline was defined as the serum potassium on the day of antibiotic initiation. The latest available measurement before initiation was chosen. If there was no measurement available at the day of initiation, measurement from the day before (second choice) or after initiation (third choice) was taken. Follow-up serum potassium was defined as the serum potassium in the period of 48–120 h after initiation. If multiple measurements were available, the highest value was chosen. The main secondary endpoint was guideline adherence, which was determined by assessing if potassium was monitoring in the high-risk groups (usage of other potassium elevating medication, age > 70, presence of heart failure, diabetes mellitus or haematological malignancies and PJP). The presence of potassium monitoring was assessed in four time slots, one before initiation of antibiotic therapy (between 1 week prior to start of antibiotic therapy) and three after initiation of antibiotic therapy (0–48 h, 48–120 h and after 120 h until the end of antibiotic therapy). Since there are no clear criteria for ‘regular’ serum potassium monitoring in the guidelines, it was decided to define guideline adherence as the presence of potassium measurement in the period of 48–120 h after antibiotic initiation. This definition was chosen because in this period, trimethoprim has reached steady state concentrations, and the potassium-sparing effects are expected to translate in an effect on serum potassium [9]. Other secondary outcomes included the incidence of hyperkalemia and change in serum creatinine (ΔCr). Hyperkalemia was defined as a serum potassium > 4.7 mmol/l at 48–120 h after initiation of antibiotic therapy. For this analysis, patients who were not normokalemic at baseline were excluded. ΔCr was determined in a similar fashion as ΔK.

All data were collected and analysed using SPSS**®** Statistics for Windows, Version 24.0 (IBM Corp. Armonk, New York, United States of America). Data are displayed as mean (± SD) for normally distributed data and median (interquartile range) for non-normally distributed data. Categorical data are represented by percentages. Cohort characteristics of both groups were compared using an independent samples t-test for numerical variables or a Pearson Chi-squared test for categorical variables. Average (change in) serum potassium and creatinine were compared using an independent samples *t*-test. A linear regression model was performed to identify and correct for potential confounders regarding change in serum potassium. The variables used in this model included antibiotic treatment, baseline potassium, age, sex, kidney dysfunction, diabetes, heart failure, haematological malignancies, kaliuretic diuretics, potassium-sparing diuretics, ACEi/ARBs, NSAIDs, corticosteroids, insulin and mortality. The incidence of hyperkalemia and the availability of serum potassium measurements were compared using Pearson Chi-squared tests.

## Results

From 223 patients who received intravenous co-trimoxazole, 167 met at least one exclusion criterion, leaving 66 patients (admission dates between May 2009 and September 2017) available for inclusion. Twenty-two of these 66 patients were included from the years 2009–2011. For these 66 patients, 132 patients using ceftriaxone were included, resulting in a total of 198 included patients.

For 36 out of the 66 included patients (55%) using co-trimoxazole, either baseline serum potassium and/or follow-up serum potassium was not available, and thus change in serum potassium could not be calculated. These 36 patients were excluded from the primary analysis, leaving 30 patients. In a similar fashion, 91 out of the 132 included patients (69%) using ceftriaxone were excluded from the primary analysis, leaving 41 patients. After data collection, 9 patients were identified (5 using ceftriaxone and 4 using co-trimoxazole) with hypokalemia at baseline (serum potassium < 3.3 mmol/l). Further investigation of the electronic patient files for serum potassium supplementation was performed and one patient on ceftriaxone who had received potassium supplementation during antibiotic therapy was excluded. Cohort characteristics were compared for the remaining 30 patients receiving co-trimoxazole [57% male, mean age 66 years (range 29–84 years)] and 40 patients receiving ceftriaxone [40% male, mean age 74 years (range 31–97 years)]. The three most prevalent indications for co-trimoxazole were PJP (47%), pneumonia or pneumosepsis (not further defined) (20%) and gastro-intestinal infections and acute diarrhea (17%). The three most prevalent indications for ceftriaxone were pneumonia or pneumosepsis (not further defined) (40%), sepsis of unknown origin or sepsis without clear focus (30%) and urinary tract infection or urosepsis (20%). The co-trimoxazole group was significantly younger (65.7 and 73.8 years, *p* = 0.012) and suffered from significantly fewer comorbidities, including kidney dysfunction (eGFR 30 to < 6) (17% and 53%, *p* = 0.003), diabetes (20% and 53%, *p* = 0.006) and heart failure (10% and 30%, *p* = 0.044). Haematological malignancies were significantly more frequent in the co-trimoxazole group (40% and 10%, *p* = 0.003). Several patients used potassium-influencing co-medication. Mortality within 2 weeks after initiation of antibiotic therapy was 17% in the co-trimoxazole and 10% in the ceftriaxone group. All cohort characteristics are displayed in Table 1.

**Table 1:** Cohort characteristics

	Co-trimoxazole	Ceftriaxone	
	*n* = *30*	*n* = *40*	
Age (years), mean ± SD	65.7 (13.4)	73.8 (12.5)	***p < 0.05***
Number of men (%)	17 (56.7)	16 (40.0)	*p* = *0.17*
Number of patients with potassium-altering comorbidities (%)			
eGFR 30 to < 60	5 (17.2)^a^	21 (52.5)	***p < 0.01***
Diabetes	6 (20.0)	21 (52.5)	***p < 0.01***
Heart failure	3 (10.0)	12 (30.0)	***p < 0.05***
Hematologic malignancies	12 (40.0)	4 (10.0)	***p < 0.01***
Number of patients taking potassium-altering drugs (%)			
Kaliuretic diuretics	11 (36.7)	18 (45.0)	*p* = *0.48*
Potassium-sparing diuretics (combined with kaliuretic diuretics)	4 (13.3)	7 (17.5)	*p* = *0.64*
Angiotensin converting enzyme inhibitors or angiotensin receptor blockers	9 (30.0)	17 (42.5)	*p* = *0.28*
Non-steroid anti-inflammatory drugs	3 (10.0)	4 (10.0)	*p* = *1.00*
Corticosteroids	15 (50.0)	9 (22.5)	***p < 0.05***
Insulin	5 (16.7)	12 (30.0)	*p* = *0.20*
Mortality (number of patients that died within 2 weeks after antibiotic therapy initiation) (%)	5 (16.7)	4 (10.0)	*p* = *0.41*

Bold and italic: independent* t*-test

Italic: Pearson Chi-squared test

^a^One patient using co-trimoxazole had no eGFR measurements and was thus excluded for the analysis of this category

**Fig. 2:**
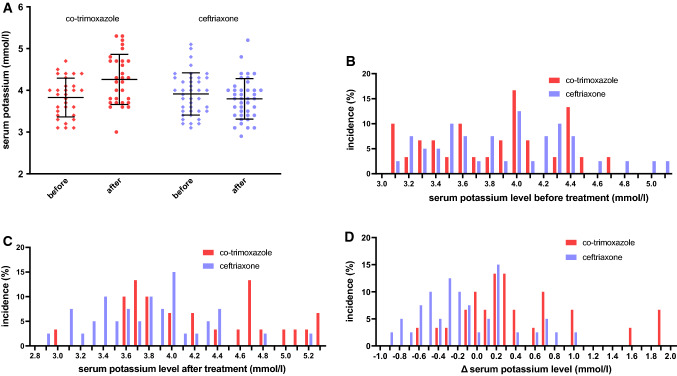
Effect of co-trimoxazole on serum potassium levels. **a** Scatter plot of serum potassium levels before and after treatment with co-trimoxazole or ceftriaxone (mean, SD). **b **Histogram showing serum potassium levels before administration of antibiotic treatment. **c** Histogram showing serum potassium levels after administration of antibiotic treatment. **d** Change in serum potassium during antibiotic treatment, defined as follow-up potassium minus baseline potassium

Baseline serum potassium was similar in both groups: 3.83 mmol/l (SD 0.46 mmol/l, range 3.1–4.7) for the co-trimoxazole group and 3.91 mmol/l (SD 0.51 mmol/l, range 3.1–5.1) in the ceftriaxone group. Follow-up potassium was on average 4.26 mmol/l (SD 0.60 mmol/l, range 3.0–5.3) in the co-trimoxazole group, 0.43 mmol/l higher than at baseline. In the ceftriaxone group, follow-up potassium was on average 3.80 mmol/l (SD 0.48 mmol/l, range 2.9–5.2), 0.11 mmol/l lower than at baseline. A rise in serum potassium was present in 22 (73%) patients using co-trimoxazole and serum potassium change ranged from − 0.6 to + 1.9 mmol/l. In the ceftriaxone group, a rise in serum potassium was present in 14 (35%) patients, and change in serum potassium ranged from − 0.9 to + 1.0 mmol/l. Change in serum potassium was significantly higher (+ 0.55 mmol/l) after co-trimoxazole, as compared to ceftriaxone (95% CI 0.29–0.80, *p* < 0.001). In Fig. 2, these data are visualized. In order to correct for potential confounders, a stepwise logistic regression model was used. Antibiotic therapy (β = 0.527, *p* < 0.001), baseline potassium (β = − 0.539, *p* < 0.001) eGFR 30 to < 60, (β = − 0.471, *p* = 0.001), the presence of haematological malignancies (β = 0.470, *p* = 0.005) and the usage of corticosteroids (β = 0.330, *p* = 0.028) were associated with a significant change in serum potassium. When corrected for these four co-variables, the effect of co-trimoxazole usage on serum potassium change decreased to 0.28 mmol/l when compared to ceftriaxone (β = 0.278, *p* = 0.031, 95% CI 0.03–0.53), but remained significant. One patient using co-trimoxazole was excluded from these linear regression models due to missing kidney function values.

Fifty-nine out of 70 (84%), were normokalemic at baseline (serum potassium 3.3–4.7 mmol/l). Six of these developed hyperkalemia at follow-up (serum potassium > 4.7 mmol/l), 4 of which used co-trimoxazole. Fifteen of normokalemic patients at baseline developed hyperkalemia in the co-trimoxazole group, compared to 6% in the ceftriaxone group (*p* = 0.239). In total, 8 patients were hyperkalemic at follow-up, 6 of which used co-trimoxazole. So, incidence of hyperkalemia was 20% in the co-trimoxazole group, compared to 5% in the ceftriaxone group. It was found that the group which received > 1920 mg of co-trimoxazole daily (n = 17) showed a 25% higher rise in serum potassium than the group using 1920 mg daily (n = 13), although this difference was not statistically significant (+ 0.48 mmol/l and + 0.36 mmol/l, *p* = 0.498). Patients using co-trimoxazole showed a mean rise of 3 µmol/l when baseline creatinine was compared to creatinine at follow-up, whereas patients using ceftriaxone saw a mean drop of 13 µmol/l (*p* = 0.001).

### Potassium monitoring

Fifty-six out of 66 (85%) patients using co-trimoxazole had a serum potassium measurement in the week before initiation, compared to 125 out of 132 (95%) patients using ceftriaxone (*p* = 0.020). In the first 48 h following the start of antibiotic therapy, 39 out of 66 (59%) patients using co-trimoxazole and 61 out of 132 (46%) patients using ceftriaxone had serum potassium monitoring (*p* = 0.088). In the period of 48–120 h into antibiotic therapy, from the 66 patients using co-trimoxazole, 36 (55%) had at least one serum potassium measurement, compared to 45 out of 132 patients using ceftriaxone (34%) (*p* = 0.006). In the period of 120 h after the start of antibiotic therapy until the end of therapy, serum potassium measurements were present in 16 out of 30 (53%) of patients using co-trimoxazole and 6 out of 23 (26%) patients using ceftriaxone (*p* = 0.046).

From the 19 patients using co-trimoxazole also using ACEi/ARBs or potassium-sparing diuretics, 13 (68%) had serum potassium measurement in the period of 48–120 h after antibiotic therapy initiation. Seventeen out of 29 (59%) patients age > 70 years and 3 out of 5 (60%) patients with heart failure, had serum potassium measurement in the period of 48–120 h after antibiotic therapy initiation. In patients with diabetes mellitus using co-trimoxazole, 6 out of 10 (60%) was monitored. In patients using NSAIDs, 4 out of 14 (29%) was monitored. For patients with haematological malignancies this concerned 15 out of 29 patients (52%). In patients using co-trimoxazole for PJP this concerned 18 out of 33 patients (55%).

## Discussion

This study showed a significant increase in serum potassium in patients using intravenous co-trimoxazole when compared to patients using intravenous ceftriaxone. There was no significantly increased incidence of hyperkalemia after co-trimoxazole compared to ceftriaxone therapy, but this may have been due to lack of power. The corrected rise of 0.28 mmol/l in serum potassium, when compared to ceftriaxone, is largely in line with previous literature, such as a recent randomized controlled trial by Chan et al., which showed an average rise of 0.21 mmol/l after 6 weeks of oral therapy [10]. Nonetheless, the observed average rise of 0.43 mmol/l (range − 0.6 to + 1.9 mmol/l) in hospitalized patients does suggest that many of these patients are at an increased risk of hyperkalemia. The observed incidence of hyperkalemia of 15–20% further underlines the importance of serum potassium monitoring in these patients. This study was not powered to investigate whether the higher incidence of hyperkalemia also translated into a higher incidence of ADRs, such as cardiac arrythmias. The observed serum potassium in our study (up to 5.3 mmol/l) may have led to additional monitoring and possibly extended hospitalisation, since potassium > 5.0 mmol/l is defined as hyperkalemia which requires adequate treatment as well as monitoring according to Dutch guidelines [9].

There are several potential limitations of this study. First, the retrospective nature means that there is a potential measuring bias. Second, information on the exact severity of disease of subjects lacks, as well as other potential potassium-influencing factors such as gastro-intestinal losses and food intake. Third, this study allows for cohort characteristics to influence its outcomes. In the linear regression model, it was found that four of the fourteen measured covariables were associated with change in serum potassium. A high baseline potassium was found to be protective for further increases in serum potassium (β = − 0.539, *p* < 0.001), as was lower kidney function (eGFR 30 to < 60; β = − 0.471, *p* = 0.001). Corticosteroid usage was also protective against rises in serum potassium (β = 0.330, *p* = 0.028). Finally, the presence of haematological malignancies was associated with higher rises in serum potassium (β = 0.470, *p* = 0.005). However, after correction of these factor, co-trimoxazole usage was still associated with significantly higher rises in serum potassium.

As for monitoring, 6 out of 19 patients who used co-trimoxazole and ACEi/ARBs and/or potassium sparing diuretics, did not have a serum potassium measurement in the time period of 48–120 h into antibiotic therapy, the period in which serum potassium is expected to reach peak values. This means that the guidelines from the Royal Dutch Pharmacists Association guidelines were not followed in 32% of cases. However, these patients were treated in 2009, 2011 (2 ×), 2013 (2 ×) and 2015, and thus partly before the studies which investigated this interaction were published. Adherence to the Dutch Pharmacotherapeutic compass, which more vaguely advises regular serum potassium measurements in ‘high-risk patients’, is hard to determine. However, serum potassium measurements in the period of 48–120 h after initiation of co-trimoxazole therapy were often missing in what the Pharmacotherapeutic compass calls ‘high-risk’ patients (e.g. age > 70 years). Most hospitalized patients that received co-trimoxazole intravenously (57 out of 66 (86%)) are considered ‘high risk’ patients for hyperkalemia by the Pharmacotherapeutic compass (meeting one or more of the following criteria: PJP diagnosis, age > 70 years, diabetes mellitus, heart failure and usage of NSAIDs, ACEi/ARBs or potassium-sparing diuretics). From these 57 patients, 33 were monitored for hyperkalemia (58%). The missing serum potassium values that were observed, may have led to hyperkalaemic events that passed unnoticed, even though there are no clear signs indicating this in the data (e.g. a higher mortality rate in the group without serum potassium monitoring using co-trimoxazole). This study showed that serum potassium measurements were significantly more often present in the co-trimoxazole group when compared to the ceftriaxone. Nonetheless, it is unclear whether this is the consequence of awareness for the potassium rising effect of co-trimoxazole, since other group differences could potentially increase the frequency serum potassium monitoring. This study does show that serum potassium measurements are far from standard in these hospitalized patients.

## Conclusion

In conclusion, intravenous co-trimoxazole usage is associated with a significant rise in serum potassium when compared to intravenous ceftriaxone and thus regular serum potassium monitoring should be considered, especially in patients at high risk for hyperkalemia. Nonetheless, serum potassium monitoring is often not performed during intravenous co-trimoxazole therapy and guideline adherence was poor. Further research is needed to determine why guideline adherence is insufficient and how this could be improved.
